# Dynamic changes of SCGN expression imply different phases of clear cell renal cell carcinoma progression

**DOI:** 10.1007/s12672-024-01071-4

**Published:** 2024-06-03

**Authors:** Tuanjie Guo, Xuan Wang, Tao Wang, Jian Zhang, Yang Liu, Siteng Chen, Xu Wang, Xiaoqun Yang, Chaofu Wang, Xiang Wang

**Affiliations:** 1 Department of Urology, Shanghai General Hospital, Shanghai Jiao Tong University School of Medicine, Shanghai, China; 2 Department of Pathology, Ruijin Hospital, Shanghai Jiao Tong University School of Medicine, Shanghai, China; 3 Department of Urology, Renji Hospital, Shanghai Jiao Tong University School of Medicine, Shanghai, China; 4 Department of Pathology, Shanghai Jiao Tong University School of Medicine, Shanghai, China

**Keywords:** SCGN, ccRCC, Occurrence, Heterogeneity, Prognosis, Biomarker

## Abstract

The secretagogin (SCGN) was originally identified as a secreted calcium-binding protein present in the cytoplasm. Recent studies have found that SCGN has a close relationship with cancer. However, its role in the occurrence, progression, and prognosis of clear cell renal cell carcinoma (ccRCC) remains unclear. In this study, we utilized a mutual authentication method based on public databases and clinical samples to determine the role of SCGN in the progression and prognosis of ccRCC. Firstly, we comprehensively analyzed the expression characteristics of SCGN in ccRCC in several public databases. Subsequently, we systematically evaluated SCGN expression on 252 microarrays of ccRCC tissues from different grades. It was found that SCGN was absent in all the normal kidney tissues and significantly overexpressed in ccRCC tumor tissues. In addition, the expression level of SCGN gradually decreased with an increase in tumor grade, and the percentage of SCGN staining positivity over 50% was 86.7% (13/15) and 73.4% (58/79) in Grade1 and Grade2, respectively, while it was only 8.3% (12/144) in Grade3, and the expression of SCGN was completely absent in Grade4 (0/14) and distant metastasis group (0/4). Additionally, the expression of SCGN was strongly correlated with the patient's prognosis, with the higher the expression levels of SCGN being associated with longer overall survival and disease-free survival of patients. In conclusion, our results suggest that reduced expression of SCGN in cancer cells is correlated with the progression and prognosis of ccRCC.

**Supplementary Information:**

The online version contains supplementary material available at 10.1007/s12672-024-01071-4.

## Introduction

Renal cell carcinoma (RCC) is the tenth most prevalent malignant tumor in the world. As a kind of cancer with wide heterogeneity, it originates from renal tubular epithelial cells and accounts for 80% of all kidney tumors [1]. Early diagnosis and successful partial or total nephrectomy can save patients' lives. However, only 10% of kidney cancer patients show characteristic clinical symptoms. More than 60% of kidney cancers are detected incidentally by ultrasound and CT examinations [2]. RCC has been classified into more than 10 subtypes based on distinctive histological features and unique molecular alterations. Notably, clear cell renal cell carcinoma (ccRCC) emerges as the predominant subtype, accounting for the highest incidence rate and serving as the primary contributor to cancer-related deaths [3]. The prognosis of ccRCC patients is still largely dependent on nuclear grade classification and clinicopathological stage. There are no biomarkers that have entered the clinic and are routinely used. Therefore, the exploration of new biomarkers is of great importance to assess the prognosis of patients and to adopt individualized treatment for different patients.

Cancer occurrence is a highly complex process caused by multiple cumulative genetic and epigenetic changes that are particularly conducive to tumor heterogeneity [4]. Intertumor heterogeneity refers to morphologic differences in the same tumor type found in different patients. Intratumor heterogeneity is the display of different histologic features within a specific tumor region. The macroscopic aspect of intertumor and intratumor heterogeneity is mainly manifested as morphological heterogeneity. We have previously demonstrated in a multicenter clinical cohort that ccRCC patients with eosinophilic features have a poorer prognosis [5]. Based on this heterogeneity, we explored microscopic heterogeneity, also known as molecular heterogeneity. We found a series of gene expression deficiencies in ccRCC patients with eosinophilic features, among which we identified the *SCGN* gene [5]. Some investigators found that SCGN deficiency leads to susceptibility to colitis [6]. SCGN is also involved in oxidized low-density lipoprotein induced endoplasmic reticulum stress and islet β-cell apoptosis [7]. SCGN overexpression promotes apoptosis and inhibits migration and invasion of human colorectal carcinoma SW480 cells [8]. It was also found that metastasis was significantly higher in the SCGN-positive subgroup of renal cell carcinoma [9]. However, the prognostic role of SCGN in ccRCC and the heterogeneous expression intratumor and intertumor are still unclear.

In this paper, we investigated the differential expression of SCGN in cancer and normal tissues and evaluated its prognostic value. We also analyzed and validated the differential expression of SCGN in ccRCC with different grades and stages according to nucleolar grade of WHO/ISUP (5th edition, 2022) and TNM classification of AJCCR (8th edition, 2018).

## Materials and methods

### Patient cohorts and data sources

In this study, we included three clinical cohorts from public databases: The Cancer Genome Atlas (TCGA) [10], Clinical Proteomic Tumor Analysis Consortium (CPTAC) [11], and E-MTAB-1980 [12]. The TCGA, CPTAC, and E-MTAB-1980 cohorts included patient survival data and RNA sequencing data. The clinical characteristics, data extraction, and processing procedures of all the patients involved in the three cohorts can be found in our previous study [13, 14]. A total of 252 patients with ccRCC from 2013 to 2022 were collected from Ruijin Hospital, and all cases were confirmed by two pathologists for diagnosis. The basic clinical characteristics of patients in the Ruijin cohort could be found in Table 1. The use of samples was approved by the ethics committee of Ruijin Hospital and the consent of the patients themselves.

**Table 1:** Basic clinical characteristics and distribution of SCGN staining of patients in the Ruijin cohort

SCGN-staining	0–25%	26–50%	51–75%	76–100%	Total (252 %)	*P*-value
Age(years)						0.344
< 65	100 (55.2)	19 (10.5)	13 (7.2)	49 (27.1)	181 (71.8)	
≥ 65	43 (60.5)	7 (9.9)	2 (2.8)	19 (26.8)	71 (28.2)	
Gender						0.004
Male	98 (65.3)	13 (8.7)	9 (6.0)	30 (20.0)	150 (59.5)	
Female	45 (44.1)	13 (12.7)	6 (5.9)	38 (37.3)	102 (40.5)	
Grade (WHO/ISUP)						< 0.001
G1	2 (13.3)	0 (0)	0 (0)	13 (86.7)	15 (6.0)	
G2	11 (13.9)	10 (12.7)	8 (10.1)	50 (63.3)	79 (31.3)	
G3	116 (80.5)	16 (11.1)	7 (4.9)	5 (3.5)	144 (57.1)	
G4	14 (100)	0 (0)	0 (0)	0 (0)	14 (5.6)	

### Cell culture

Human ccRCC cell lines 786-O, Caki-1 and A498 were obtained from the ATCC (American Type Culture Collection) and were cultured in DMEM (Dulbecco's Modified Eagle Medium) (Product No. 11965092, Thermo Fisher, United States). HK-2 cells were obtained from the ATCC and were cultured in DMEM-F12 (Dulbecco’s Modified Eagle’s Medium/Nutrient Mixture F-12) (Product No. D0697, Sigma-Aldrich, Germany). All the cell lines were supplemented with 100 U/ml streptomycin/penicillin (Product No. 15140148, Thermo Fisher, United States) and 10% FBS (Fetal Bovine Serum) (Product No. A5669701, Sigma-Aldrich, Germany) at 37 °C in a humidified atmosphere of 5% CO_2_. All of the cells were tested to ensure that they are *Mycoplasma* free. All cells were cultured on six-well plates (Product No. 3516, Corning, United States).

### Tissue microarray and immunohistochemistry

Tissue microarrays (TMAs) were constructed using a tissue microarrayer (Manual tissue microarrayer, UNITMA). One 15 mm core was collected from each patient. All lesions were reevaluated based on WHO/ISUP 2022 nucleolar grade. Haematoxylin and eosin (H and E)–stained and Immunohistochemical (IHC) analyses were performed on 4 μm formalin-fixed paraffin-embedded (FFPE) tissue sections with antibodies reactive for the following antigens: SCGN (Product No. A19615, ABclonal, China, 1:150). The nuclear staining of the tumor cells was assessed as positive, and the investigators who reviewed the slides independently were blinded to the final diagnosis. The proportion of the staining-positive cells was divided into 4 groups: 0–25%, 26–50%, 51–75%, and 76–100%.

### Western blotting

Cells were rinsed with 1 × ice-cold PBS (Product No. 10010023, Thermo Fisher, United States) before lysis in radioimmunoprecipitation assay buffer (RIPA) (Product No. 10010023, Thermo Fisher, United States) on ice. The RIPA lysis buffer contained 1 × protease and phosphatase inhibitor (Product No. P1005, Beyotime, China). After centrifugation at 4 °C for 20 min at 20000 g, protein lysates were quantified using the BCA Protein Assay Kit (Product No. P0010, Beyotime, China) as per the manufacturer’s instructions, and absorbance was measured with a GloMax Discover Microplate Reader (Promega, United States). Subsequently, protein samples were mixed with 5 × sodium dodecyl sulfate (SDS) loading buffer (Product No. P0015L, Beyotime, China) and boiled at 95 °C for 10 min. The proteins underwent separation using 10% sodium dodecyl sulfate–polyacrylamide gel electrophoresis (SDS-PAGE) gels (Product No. PG112, Shanghai Epizyme Biomedical Technology, China) and transfer to a PVDF membrane (Product No. IPVH00010, Millipore, Germany). After blocking with Tris-buffered saline with 0.1% Tween 20 detergent (Product No. ST671, Beyotime, TBST) supplemented with 5% nonfat milk for 60 min at room temperature, the membrane was rocked before overnight blotting with primary antibodies at 4 °C. Subsequent steps involved washing the membrane in TBST three times for 5 min each and blotting in secondary antibodies at room temperature for 1 h. Tanon 4600SF Chemiluminescent Image Analysis System (Tanon, China) was used to visualize the membrane. The antibodies used were anti-SCGN (Product No. A19615, ABclonal, China, 1:1000), anti-β-actin (Product No. AC038, ABclonal, China, 1:20,000), and HRP Goat Anti-Rabbit IgG (Product No. AS014, ABclonal, China, 1:20,000).

### Statistical analysis

Continuous variables were compared using the Mann–Whitney U test and Student’s t-test. For more than two groups, analysis of variance (ANOVA) was employed. The chi-square test was used to compare the proportion of clinical characteristics. Kaplan–Meier curve analysis, along with the log-rank test, was conducted to compare overall survival (OS) and disease-free survival (DFS).Part of the survival analysis was performed through GEPIA [15]. All statistical analyses and visualization were performed in R (4.0.0), SPSS (26), GraphPad Prism (8), and MedCalc (20.113).

## Results

### SCGN transcript levels are elevated in ccRCC

**Fig. 1:**
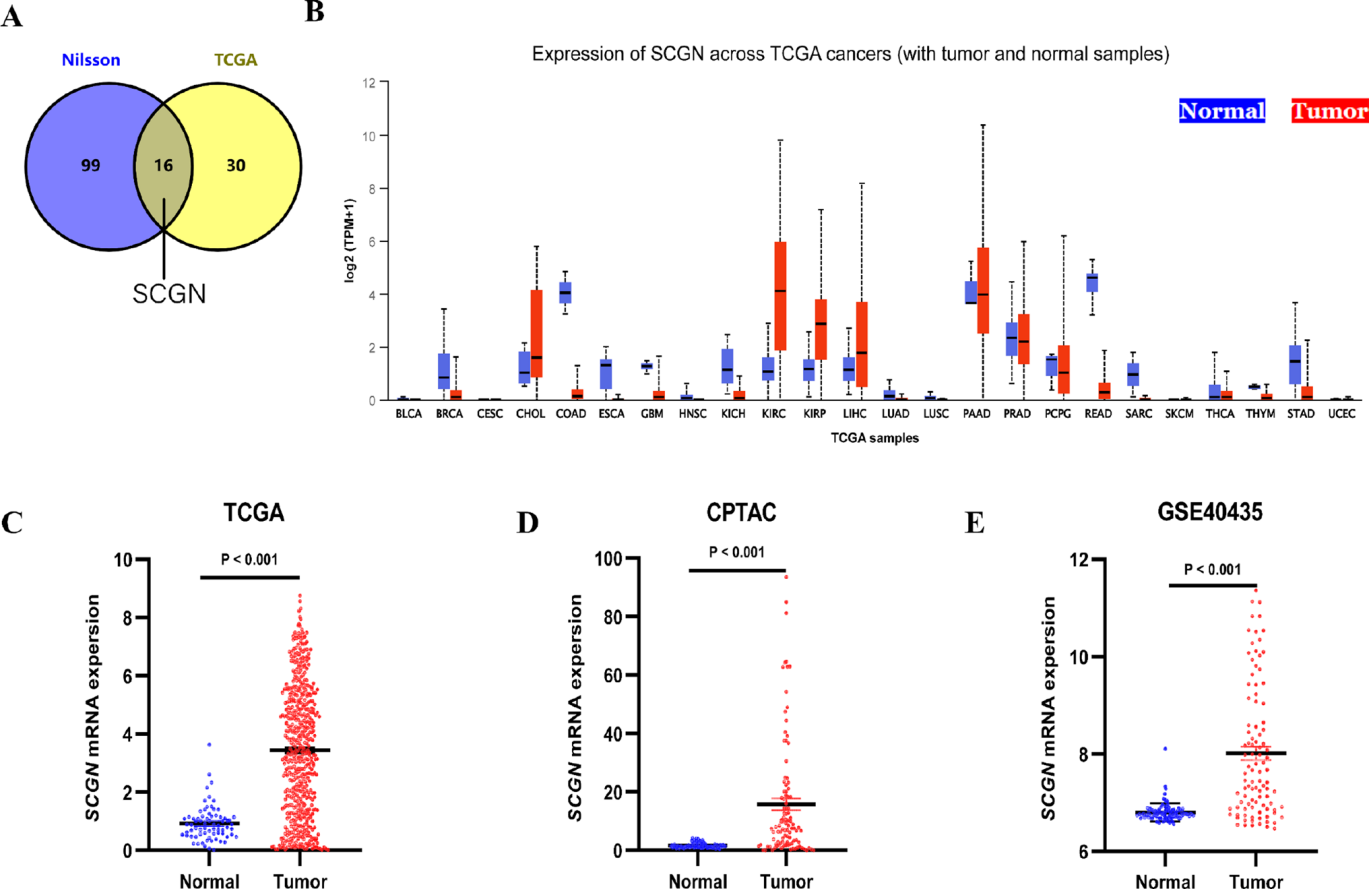
SCGN mRNA levels are upregulated in ccRCC. **A** A Venn diagram: the origin of SCGN. **B** Expression of SCGN mRNA in pan-cancer. **C** Comparison of transcript levels of SCGN in cancer and normal tissues in the TCGA cohort. **D** Comparison of transcript levels of SCGN in cancer and normal tissues in the CPTAC cohort. **E** Comparison of transcript levels of SCGN in cancer and normal tissues in the GSE40435 cohort

Previously, when performing differential expression gene analysis between eosinophilic and clear ccRCC in the TCGA cohort, we identified 46 genes that were significantly downregulated in eosinophilic ccRCC [5]. We then took the intersection with the RNA sequencing results of Nilsson et al. [16]. In the intersection, we found SCGN (Fig. 1A). To visualize the expression level of SCGN in pan-cancer, we performed a pan-cancer analysis of SCGN expression level in the TCGA database using UALCAN [17]. It shows that SCGN has almost the highest expression level in ccRCC and also has the greatest difference compared to normal tissues (Fig. 1B). To fully illustrate and confirm the differential expression of SCGN in ccRCCs and normal kidney tissues, we analyzed RNA sequencing data using multiple databases, and we obtained consistent results in TCGA, CPTAC and GSE40435 cohorts that the transcript levels of SCGN were higher in ccRCC than in normal kidney (Fig. 1C–E).

### SCGN protein is upregulated in ccRCC

**Fig. 2:**
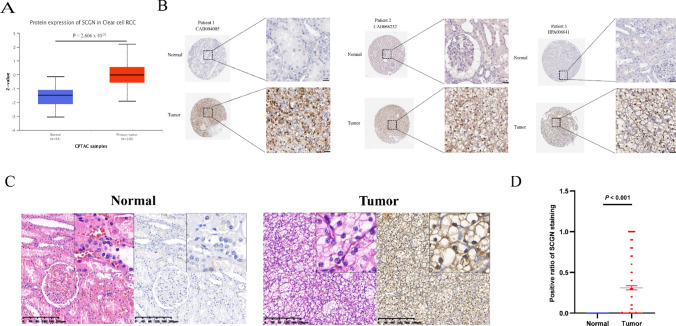
SCGN protein is upregulated in ccRCC. **A** Comparison of SCGN expression in cancer and normal tissues in the CPTAC database. **B** IHC of SCGN in cancer and normal tissues in the HPA database. **C** IHC of SCGN in cancer and normal tissues in our cohort. **D** Nuclear positive ratio statistical analysis of cancers and normal tissues under IHC in our cohort

We next explored whether the protein levels of SCGN were higher in tumors than in normal kidney tissues. We initially analyzed the difference in protein expression of SCGN in cancer and normal tissues in the CPTAC database using the UALCAN website. As shown in Fig. 2A, the translation level of SCGN was remarkably higher in ccRCC than in normal kidneys. Afterward, we confirmed the differential expression of SCGN in normal kidney tissues and tumors from the IHC perspective in the HPA database. IHC results from three patients from the HPA database showed that SCGN was expressed at dramatically higher levels in the tumors of ccRCC than in normal tissues and that SCGN protein was barely detectable in normal kidney tissues (Fig. 2B). SCGN was significantly highly expressed in the carcinomas and was heavily concentrated in the nucleus. We verified this result in our cohort (Fig. 2C). Our statistical analysis of tissue microarrays reinforced the fact that SCGN was highly expressed in ccRCC (Fig. 2D). Among the different patients, the distribution and number of cases of SCGN positivity were 0–25% (143, 56.7%) 26–50% (26, 10.3%), 51–75% (15, 6.0%), and 76–100% (68, 27.0%), respectively (Table 1).

### SCGN expression is absent in high-grade ccRCC

**Fig. 3:**
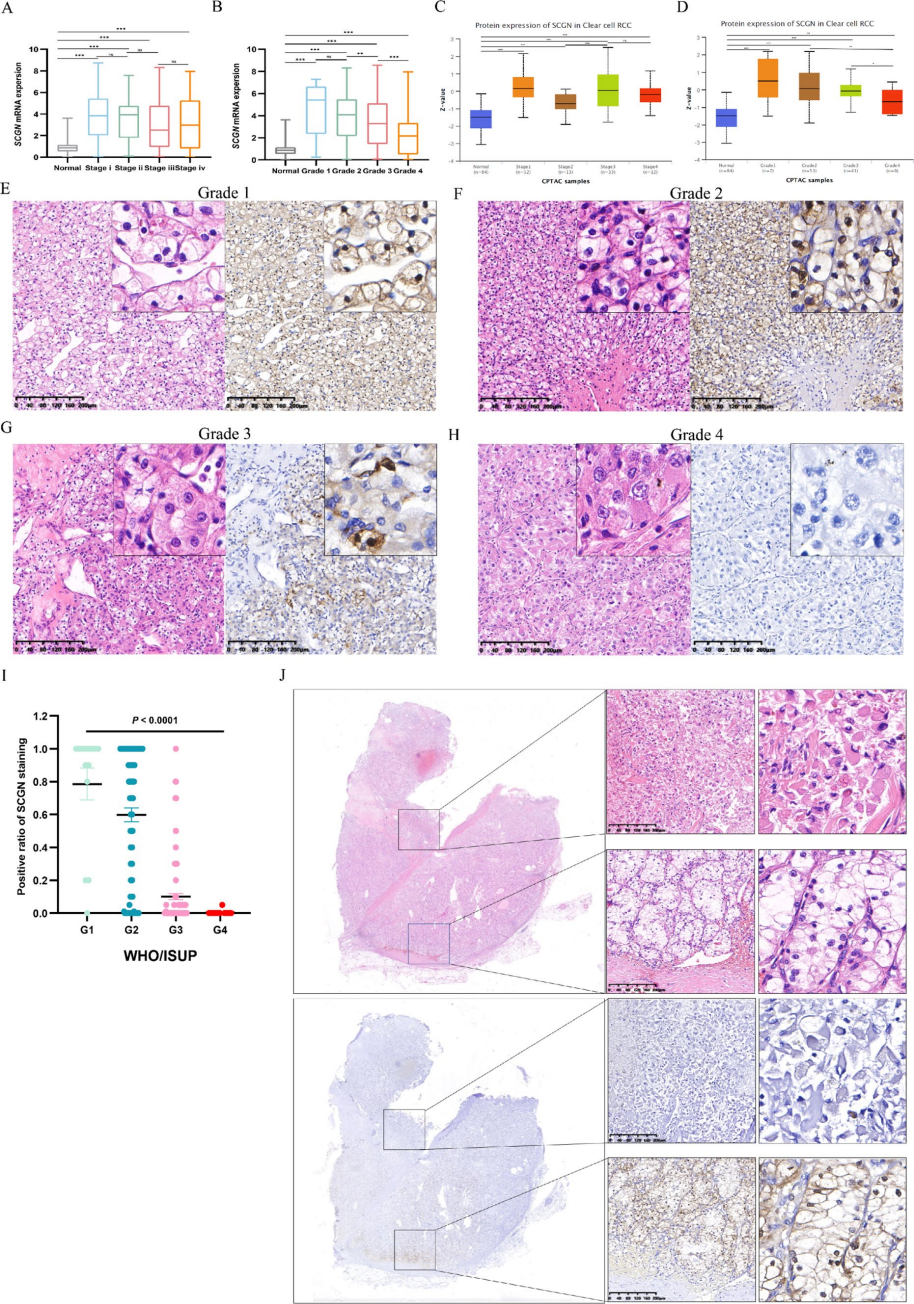
As ccRCC progressed SCGN expression was gradually decreased. **A** The relationship between SCGN mRNA level and stages in TCGA cohort. **B** The relationship between SCGN mRNA level and WHO/ISUP nuclear grades in TCGA cohort. **C** The relationship between SCGN protein level and stages in CPTAC cohort. **D** The relationship between SCGN protein level and WHO/ISUP nuclear grades in CPTAC cohort. **E** HE and IHC of SCGN of ccRCC patients with nuclear grade 1. **F** HE and IHC of SCGN of ccRCC patients with nuclear grade 2. **G** HE and IHC of SCGN of ccRCC patients with nuclear grade 3. **H** HE and IHC of SCGN of ccRCC patients with nuclear grade 4. **I** Statistical analysis of nuclear positive ratio of tissue microarrays between different ccRCC grades. **J** Intratumoral heterogeneous expression of SCGN in ccRCC

Next, we explored whether SCGN expression was inextricably linked to the ccRCC stage and WHO/ISUP nucleolar grade. First of all, we compared the mRNA expression of SCGN between different stages in the TCGA database. Although there was no significant statistical difference, it was still obvious that the expression of SCGN gradually decreased as the stage increased (Fig. 3A). We then compared the mRNA expression levels of SCGN in patients with different WHO/ISUP nucleolar grades, and SCGN showed a significant decreasing trend with increasing nuclear grade (Fig. 3B). To increase the reliability of the previous conclusions, we repeated the previous comparisons using the CPTAC database at the protein level of SCGN. Encouragingly, we obtained the same conclusion when ccRCC progressed and nuclear grade was elevated, the expression of SCGN at the protein level gradually decreased (Fig. 3C–D). We verified this finding by IHC staining of SCGN in ccRCC patients with different nuclear grades (Fig. 3E–H). SCGN is almost undetectable when the nuclear grades are at grade 3 and 4. Furthermore, this finding was also supported by our tissue microarray data (Fig. 3I). Considering ccRCC heterogeneity, all TMA tumor tissues were re-scored for WHO/ISUP nuclear grade. The percentages of SCGN staining positivity over 50% were 86.7% (13/15) and 73.4% (58/79) in Grade1 and Grade2, respectively, while it was only 8.3% (12/144) in Grade3, and the expression of SCGN was completely absent in Grade4 (0/14). The distribution of SCGN IHC staining results among different grades were summarized in Table 1. Subsequent evaluation of panoramic tissue sections revealed that the staining of SCGN varied with nuclear grade in different regions of the same tumor. We found high SCGN expression in areas of low WHO/ISUP nucleolar grade and almost no SCGN expression in areas of high WHO/ISUP nucleolar grade (Fig. 3J).

### SCGN deficiency is linked to distant metastasis of ccRCC

**Fig. 4:**
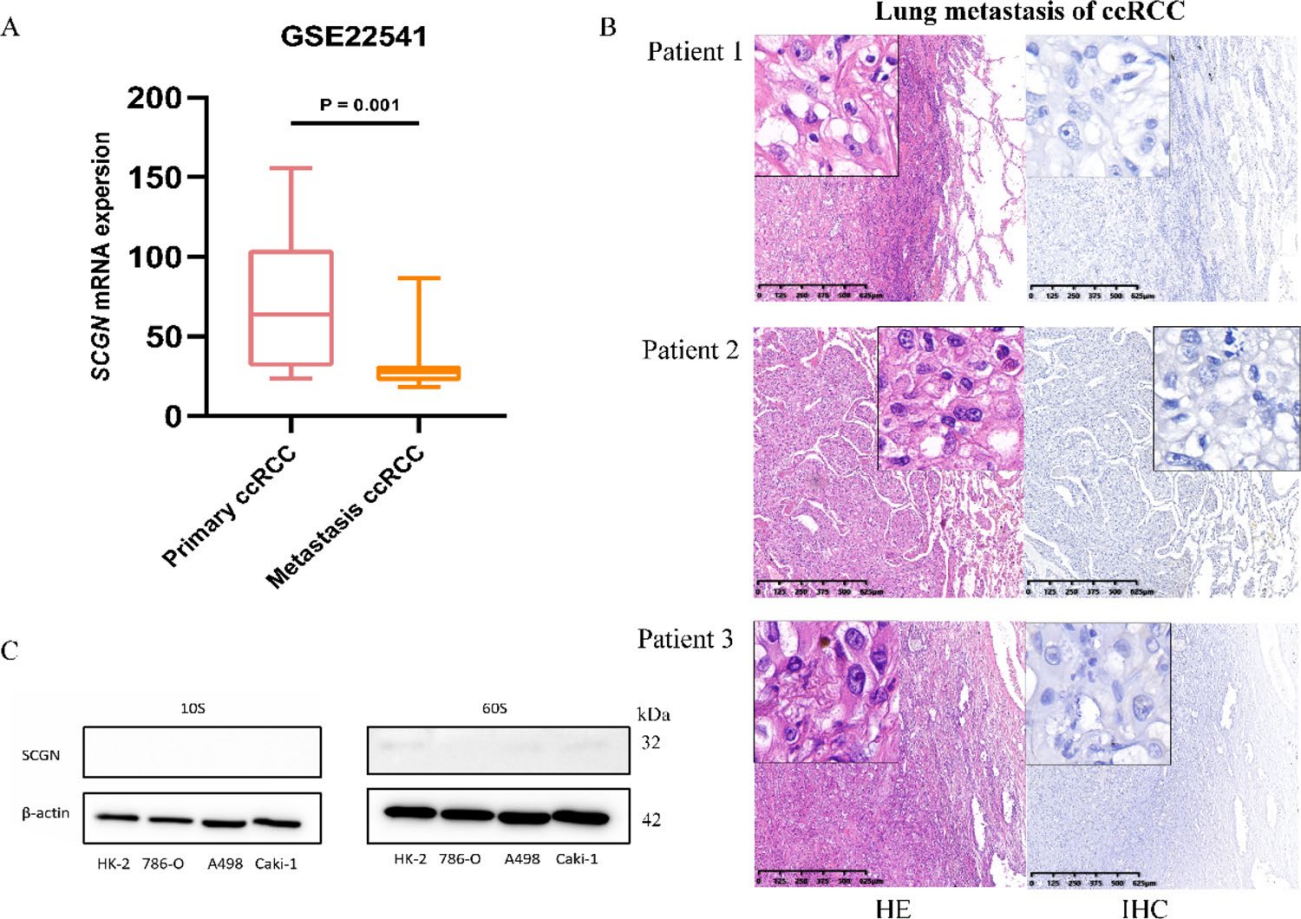
Absence of SCGN expression in distant metastases of ccRCC. **A** Comparison of SCGN expression between primary and distant metastatic foci in ccRCC. **B** HE and IHC of ccRCC with lung metastasis. **C** Comparison of SCGN expression between cell lines of ccRCC

Since the expression of SCGN is gradually decreased with tumor progression, we wanted to know the expression status of SCGN in metastatic tumors of ccRCC. Firstly, we compared the difference of mRNA expression of SCGN in primary and metastatic tumors in GSE22541 cohort. We found that the expression of SCGN was lower in metastatic sites compared with the primary sites (Fig. 4A). We then performed IHC in three cases of ccRCC with lung metastasis, all three cases are Grade 3 or higher nuclear grade and found that SCGN was not detected in the metastasis of ccRCC (Fig. 4B). We examined the expression of SCGN in immortalized renal tubular epithelial cell line HK-2, primary ccRCC cell lines 786-O and A498, and skin-metastatic ccRCC cell line Caki-1, and found that SCGN was barely detectable. This is consistent with our previous findings. On one hand, there was no SCGN expression in normal kidney tissues, and on the other hand, there was almost no SCGN expression in ccRCC with higher malignancy and metastatic foci (Fig. 4C). Collectively, these results suggested that the absence of SCGN was associated with distant metastasis of ccRCC.

### Patients with high SCGN expression have a better clinical outcome

**Fig. 5:**
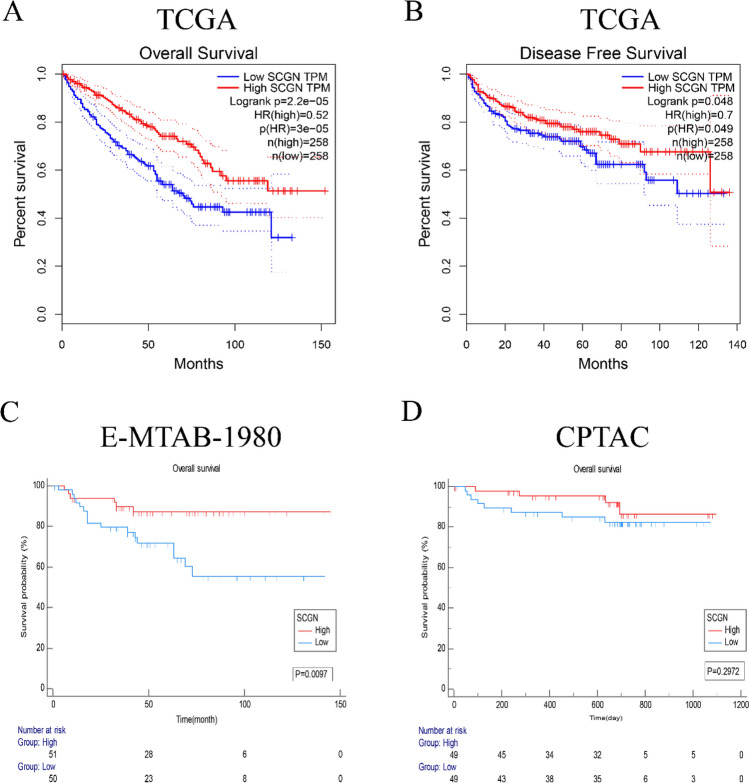
Patients with high SCGN expression have a longer survival time. **A**–**B** In the TCGA cohort, patients were grouped according to SCGN expression to compare OS and DFS. **C** In the E-MEAB-1980 cohort, patients were grouped according to SCGN expression to compare OS. **D** In the CPTAC cohort, patients were grouped according to SCGN expression to compare OS

Then, we studied whether the expression level of SCGN was related to the survival of patients. At first, we divided the patients into high and low-expression groups according to the median SCGN expression in the TCGA database, and subsequently compared the OS and DFS of the two groups. Patients in the SCGN high-expression group had longer OS and DFS than those in the SCGN low-expression group (Fig. 5A–B). We obtained similar results in the E-MTAB-1980 cohort (Fig. 5C). In the CPTAC cohort, although there was no statistical difference in OS between the two groups, a similar trend was observed, which might be ascribed to the short follow-up period of patients in this cohort (Fig. 5D). Subgroup clinical analysis demonstrated that SCGN also has a significant prognostic value at different stages of tumor progression (Fig. S1A–K). Since there were differences in SCGN staining between genders (Table 1), we analyzed whether SCGN expression affected overall survival in patients of different genders. As a result, we found that SCGN also had a prognostic value between the different genders (Fig.S2A–B).

### SCGN can be used as an independent prognostic factor for ccRCC patients

Finally, we wanted to know whether SCGN can be used as an independent prognostic factor for ccRCC patients. To achieve this, we performed univariate and multivariate Cox regression analysis in the TCGA cohort. Multivariate Cox regression analysis showed that SCGN could be used as an independent prognostic factor (Table 2). To enable a more intuitive assessment of patient prognosis using SCGN, we constructed the Nomogram prediction model (Fig. S3A). We evaluated the predictive efficacy of the Nomogram prognostic model. The result demonstrated that the prognostic model has strong robustness (Fig. S3B-C). Overall, our findings suggest that SCGN is a valuable biomarker for evaluating the prognosis of ccRCC patients.

**Table 2:** Univariate and multivariate Cox regression analysis in the TCGA cohort

Characteristics	Univariate analysis		Multivariate analysis	
	HR (95% CI)	p-Value	HR (95% CI)	p-Value
Age	1.028 (1.015–1.041)	< 0.001	1.031 (1.017–1.046)	< 0.001
Gender	0.969 (0.709–1.323)	0.841		
Grade	2.307 (1.882–2.827)	< 0.001	1.416 (1.125–1.782)	0.003
Stage	1.880 (1.647–2.145)	< 0.001	1.681 (1.446–1.954)	< 0.001
SCGN	0.852 (0.793–0.915)	< 0.001	0.899 (0.835–0.967)	0.004

## Discussion

ccRCC is a kind of cancer with high molecular and histological heterogeneity [18]. It is of great significance to explore molecular markers that can distinguish its heterogeneity. Our previous work in identifying molecular differences between eosinophilic and clear ccRCC revealed a significant difference in SCGN expression between the two ccRCC subtypes [5], which stimulated our interest in further investigating the role of SCGN in ccRCC development and progression. In the present study, by using public databases and experimental data to validate each other, we first found that SCGN was not expressed in normal kidney tissues, however, it was strongly positively expressed in ccRCC. It was further revealed that the expression of SCGN gradually decreased as the stages and grades increased. SCGN expression was deficient in tumors at metastatic sites of ccRCC. SCGN expression was heterogeneous within tumors of the same patient, that is, low expression in sites with high malignancy and high expression in sites with low malignancy. Finally, we also discovered that SCGN could be used as a prognostic marker for the prognosis of ccRCC patients.

The main finding of this study is that the expression level of SCGN alters with the progression of ccRCC, with the highest expression at the beginning of tumor formation and decreases with increasing malignancy and metastasis, and it is also heterogeneously expressed in different malignant regions within the same tumor. No similar markers have been reported until now [19, 20]. To the best of our knowledge, SCGN is one of the few biomarkers that can be used for the dynamic evolution and heterogeneity of ccRCC. In addition, it is a sufficient indication that SCGN plays a very important role in indicating the malignant transformation of ccRCC.

Secretagogin (SCGN) is a calcium-binding protein belonging to the Hexa EF-hand family, which was first identified in the cDNA library of human pancreatic β-cells and has a role in promoting insulin secretion [21, 22]. Recent studies have found that SCGN can regulate insulin signaling by directly binding insulin and also acts as a Ca^2+^-dependent stress-responsive chaperone [22, 23]. Additionally, SCGN exhibits higher affinity to Ca^2+^ in a reducing milieu and shows greater stability in an oxidizing environment [24]. SCGN also has a very significant diagnostic value in neuroendocrine types of carcinomas [25, 26]. The insulin signaling pathway not only regulates blood glucose but also promotes neovascularization in tumors, cancer cell proliferation, and cancer progression and metastasis [27]. However, clinically, the level of insulin receptor (IR) expression was significantly lower in renal cell carcinoma tissues of patients with tumor stage pT2-4 and/or distant metastases. High IR expression levels were significantly associated with better disease-free survival and overall survival after nephrectomy [28]. This is consistent with the findings of this study, suggesting that SCGN and insulin-related signaling pathways are inextricably linked in ccRCC and need to be further investigated in depth.

SCGN was initially identified between eosinophilic and clear ccRCC. The eosinophilic ccRCC is featured with more mitochondria and fewer lipid droplets in the cytoplasm, and the clear type of ccRCC is characterized by more lipid droplets and fewer mitochondria in the cytoplasm of cancer cells [16]. Mechanistically, the deficiency of SCGN might lead to the upregulation of genes related to mitochondrial production and fatty acid β-oxidation, resulting in the catabolism of intracellular lipid components. It is also possible that the absence of SCGN leads to a decrease in lipid production in cancer cells, resulting in a relative increase in lipid droplets and mitochondria. Therefore, the focus of subsequent studies should be to explore whether the absence of SCGN leads to the loss of lipid droplets and the increase of mitochondria in cancer cells. In addition, whether SCGN deficiency promotes the occurrence of epithelial-mesenchymal transition in ccRCC and confers the ability of cancer cells to metastasize distantly also deserves further in-depth investigation.

The limitation of this study lies in the lack of validation and comparison of mRNA expression levels of SCGN in clinical samples. Instead, multiple public datasets were utilized to corroborate the findings. Additionally, the significant heterogeneity of tumors can result in varying nuclear grades of tumor cells within different regions of the same tumor. As a result, the nuclear grade of selected tumor regions was reevaluated in all tissue microarrays (TMAs) included in our study. However, the limited area covered by TMAs prevented the evaluation of the relationship between the percentage of positive SCGN expression and prognosis in all tumors. Comparison of paired SCGN expression is lacking because the primary tumors and metastases of ccRCC patients were not resected simultaneously. The prognostic analysis of SCGN was relied on mRNA levels from public databases, while no survival analysis at the protein level was conducted using our clinical cohort due to the lack of follow-up records for most patients. The mechanisms of how SCGN affects patient prognosis were not further investigated in this study.

## Conclusions

In summary, we verified the role of SCGN in the occurrence, progression, heterogeneity, and prognosis of ccRCC through a combination of public databases and experimental data in this study. We found SCGN is expected to be a biomarker of ccRCC occurrence, prognosis, and progression. Furthermore, SCGN has the potential to be used as a biomarker for routine detection in clinicopathology to assess tumor heterogeneity and patient’s prognosis. In addition, the mechanism of SCGN inhibiting the progression of ccRCC needs to be further explored.

### Supplementary Information

Additional file1 (DOCX 816 KB)

## Data Availability

This study included three public datasets, which are the TCGA (https://portal.gdc.cancer.gov/), E-MTAB-1980 (https://www.ebi.ac.uk/), CPTAC (https://pdc.cancer.gov/pdc/). The datasets used and analyzed in this study are also available from the corresponding author upon reasonable request.

## Abbreviations

RCC: Renal cell carcinoma
ccRCC: Clear cell renal cell carcinoma
TCGA: The Cancer Genome Atlas
CPTAC: Clinical Proteomic Tumor Analysis Consortium
HE: Hematoxylin–eosin
TMAs: Tissue microarrays
IHC: Immunohistochemical
FFPE: Formalin-fixed paraffin-embedded
ANOVA: Analysis of variance
OS: Overall survival
DFS: Disease-free survival
