# Supplementary material for: Dynamic changes of SCGN expression imply different phases of clear cell renal cell carcinoma progression
Source: Discov Oncol. 2024 Jun 3;15:205. doi: 10.1007/s12672-024-01071-4 (PMC11147981; doi:10.1007/s12672-024-01071-4)
Supplement: Supplementary file 1 — Additional file1 (DOCX 816 KB) [file 12672_2024_1071_MOESM1_ESM.docx]

**Supplementary Figures**


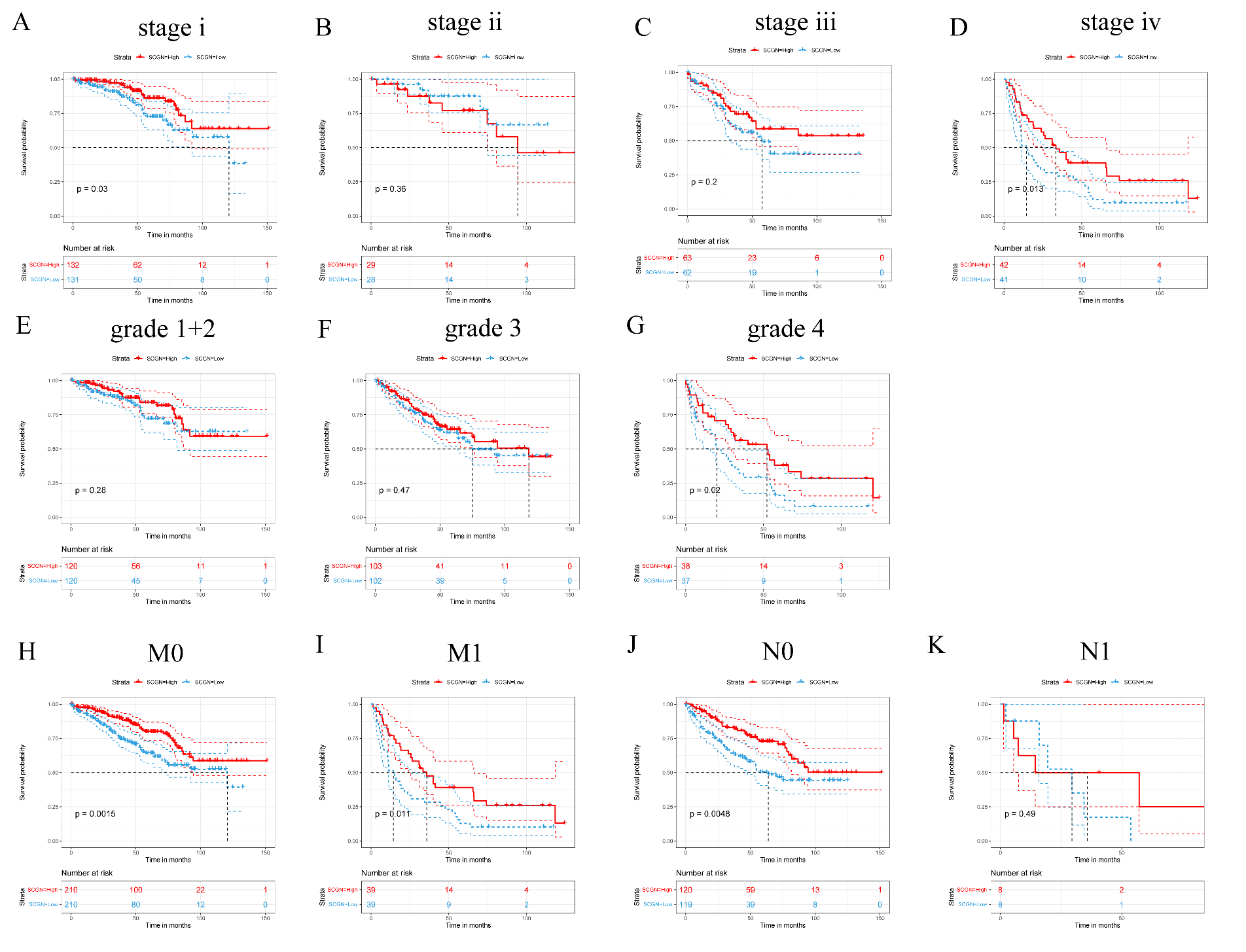


**Figure S1** Subgroup survival analysis. (A-K) In the TCGA cohort, patients were grouped according to their clinical characteristics and SCGN expression to compare OS.


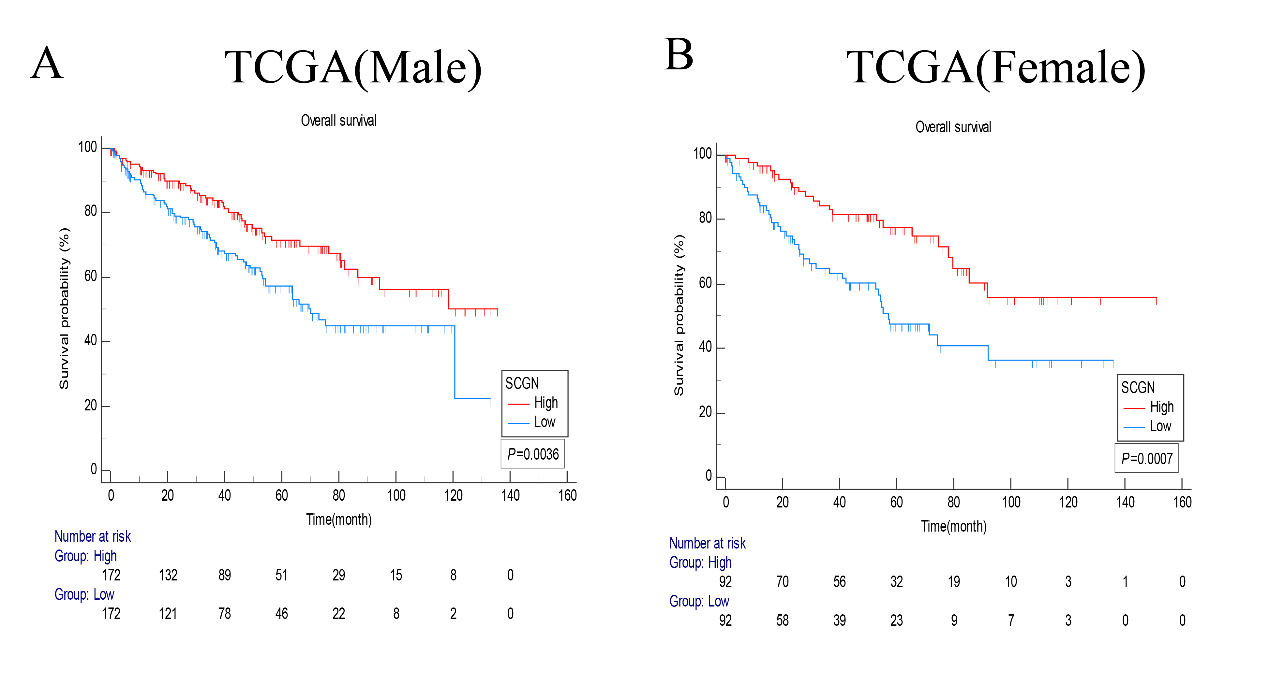


**Figure S2. Survival analysis between different genders and SCGN expression levels.**


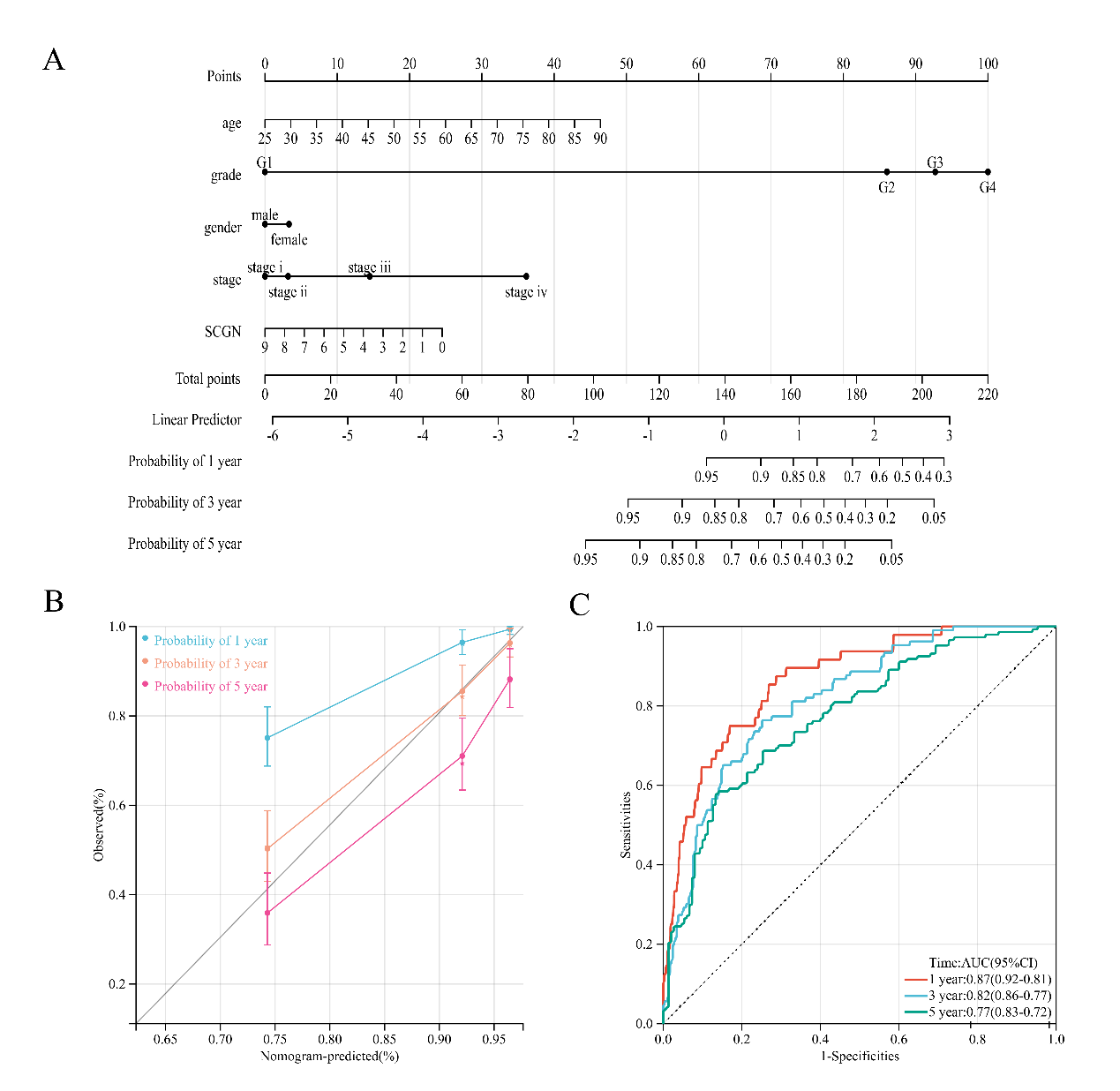


**Figure S3. The Nomogram prediction model based on SCGN. (A) Nomogram construction based on SCGN and clinical features. (B) Prediction efficiency of nomogram. (C) ROC curves for survival time of ccRCC patients based on SCGN expression.**
